# Perioperative individualized nursing for an adolescent with recurrent tracheoesophageal fistula after endoscopic repair: a case report

**DOI:** 10.3389/fped.2026.1958428

**Published:** 2026-10-23

**Authors:** Menghua Xia, Zheng Tan, Jia Liu, Lei Wu, Hangyan Zhao, Jie Xia

**Affiliations:** 1 Anesthesiology and Operation Center, Children's Hospital, Zhejiang University School of Medicine, National Clinical Research Center for Children and Adolescents' Health and Diseases, Hangzhou, Zhejiang, China; 2 Department of Thoracic Surgery, Children's Hospital, Zhejiang University School of Medicine, National Clinical Research Center for Children and Adolescents' Health and Diseases, Hangzhou, Zhejiang, China; 3 Department of ENT and Head & Neck Surgery, Children's Hospital, Zhejiang University School of Medicine, National Clinical Research Center for Children and Adolescents' Health and Diseases, Hangzhou, Zhejiang, China; 4 Endoscopy Center, Children's Hospital, Zhejiang University School of Medicine, National Clinical Research Center for Children and Adolescents' Health and Diseases, Hangzhou, Zhejiang, China

**Keywords:** case report, flexible bronchoscopy, perioperative nursing, recurrent tracheoesophageal fistula, suspension laryngoscopy

## Abstract

**Background:**

Congenital esophageal atresia (CEA) type III is a common neonatal digestive malformation, and recurrent tracheoesophageal fistula (rTEF) is a refractory long-term postoperative complication. Combined suspension laryngoscopy and flexible bronchoscopy minimally invasive fistula repair is a novel therapeutic strategy, yet standardized perioperative nursing experience targeting adolescents with multiple failed transthoracic surgeries remains scarce. Conventional nursing cannot simultaneously manage compound risks including severe malnutrition, tracheomalacia, adolescent anxiety and airway edema. This case report aimed to summarize multidisciplinary team (MDT) individualized full-cycle nursing protocols for this rare patient population.

**Case presentation:**

A 17.5-year-old male patient with rTEF, 17.5 years after neonatal primary congenital esophageal atresia repair, with two failed transthoracic fistula closures, presented persistent feeding choking, recurrent pneumonia and severe malnutrition (BMI=15.5 kg/m^2^). MDT refined airway management, quantitative staged nutrition, whole-course psychological intervention and predictive complication prevention were implemented before and after endoscopic fistula repair. No severe complications occurred during hospitalization. Esophagography and bronchoscopy on postoperative day 10 confirmed complete fistula healing. The patient was discharged on postoperative day 14 with a 0.8 kg weight gain, and one-month follow-up showed sustained fistula repair and continuous weight improvement.

**Conclusion:**

MDT-driven individualized perioperative nursing effectively reduces airway and nutritional complications and promotes fistula healing in adolescent rTEF patients receiving endoscopic minimally invasive surgery. This case provides replicable evidence-based nursing references for similar complex rare esophageal malformation cases.

## Introduction

Congenital Esophageal Atresia (CEA) is a severe congenital gastrointestinal malformation in neonates, with an incidence of 2–4 cases per 10,000 live births (1). Approximately 70% to 90% of patients present with Type III CEA, which is characterized by atresia of the upper esophagus and fistula formation between the lower esophagus and trachea, requiring early surgical intervention (2, 3). Despite substantial progress in surgical techniques including thoracoscopic and Da Vinci robotic surgery, a variety of long-term postoperative complications may still occur. Among these complications, recurrent tracheoesophageal fistula (rTEF) remains a challenging clinical issue (4, 5). Recurrent TEF often leads to recurrent pulmonary infection, feeding choking and malnutrition, which seriously compromise patients' quality of life (6).

In recent years, combined suspension laryngoscopy and flexible bronchoscopy transtracheal fistula suture has become a preferred minimally invasive alternative for patients with recurrent fistula after multiple thoracic surgeries, avoiding secondary large thoracic trauma (7, 8)^.^ This endoscopic approach requires strict perioperative airway protection and precise nutritional intervention, yet relevant systematic nursing reports are limited to infant cohorts (9). Adolescent patients with a decade-long disease course and multiple surgical failures face unique nursing challenges: severe malnutrition-induced poor tissue repair capacity, tracheomalacia secondary to long-term reflux stimulation, and prominent adolescent anxiety and low self-esteem. Conventional fragmented single-discipline nursing cannot simultaneously address these compound risks.

Multidisciplinary collaborative management integrates surgical, endoscopic, nutritional, psychological and rehabilitation resources to deliver personalized full-cycle intervention, which has been proven effective in managing complex pediatric digestive malformations (10). However, standardized quantitative nursing procedures targeting adolescent rTEF patients receiving dual-endoscopic repair have not been fully documented in current literature. This case report outlines the complete perioperative multidisciplinary team (MDT) nursing process of a 17.5-year-old male with refractory rTEF, which will be presented using the 2013 CARE checklist (11).

## Case presentation

### Patient information

The patient was a male adolescent aged 17 years and 6 months. On admission, his height was 178 cm, body weight 49.2 kg, and body mass index (BMI) 15.5 kg/m^2^, presenting an emaciated appearance.

During the neonatal period, he was diagnosed with Gross type III congenital esophageal atresia combined with tracheoesophageal fistula (TEF) due to severe feeding intolerance. Open transthoracic esophageal end-to-end anastomosis plus ligation and repair of tracheoesophageal fistula was performed on postnatal day 3. Esophagography performed on postoperative day 10 confirmed satisfactory esophageal healing, and oral feeding was initiated thereafter. However, feeding-associated choking developed at postoperative week 2, and follow-up esophagography verified recurrent TEF. His feeding regimen was switched to nasogastric tube feeding, after which he achieved steady weight gain with only occasional choking episodes, and he was discharged subsequently. After discharge, the patient maintained normal physical growth with appropriate gains in height and weight. Follow-up esophagography conducted 3 months postoperatively revealed no obvious fistulous leakage, which was considered a false-negative result potentially caused by spontaneous shrinkage of the fistula tract. No further hospital follow-up was completed afterwards.

In January 2024, the patient developed severe pneumonia accompanied by feeding choking secondary to fever during school attendance, and he was readmitted to our hospital. Esophagography and flexible bronchoscopy confirmed persistent recurrent TEF (Figure 1). After his respiratory condition was stabilized by antibiotic therapy therapy, gastroscopy-assisted TEF closure with four titanium clips(Endo-therapy rotatable clips; Olympus Medical Systems Corp, Tokyo, Japan) was performed(Figure 2). Esophagography at postoperative day 10 showed complete obliteration of the fistula; the patient tolerated oral feeding without discomfort and was discharged. In February 2025, cross-infection at school triggered recurrent pneumonia that responded poorly to antibiotic treatment. After repeated communication with his guardians, a second open transthoracic TEF repair was carried out. Postoperatively, persistent cough, sputum production and feeding choking persisted, and follow-up esophagography still indicated residual patent tracheoesophageal fistula, mainly attributed to the fact that only esophageal orifice was closed in prior intervention while tracheal-side opening remained unmanaged. His clinical symptoms improved with comprehensive antibiotic therapy and nutritional support before discharge. Post-discharge follow-up documented recurrent respiratory tract infections with unsatisfactory responses to antibiotics, as well as progressive deterioration of nutritional status. A detailed timeline of the patient's clinical course is provided in Table 1.

**Figure 1:**
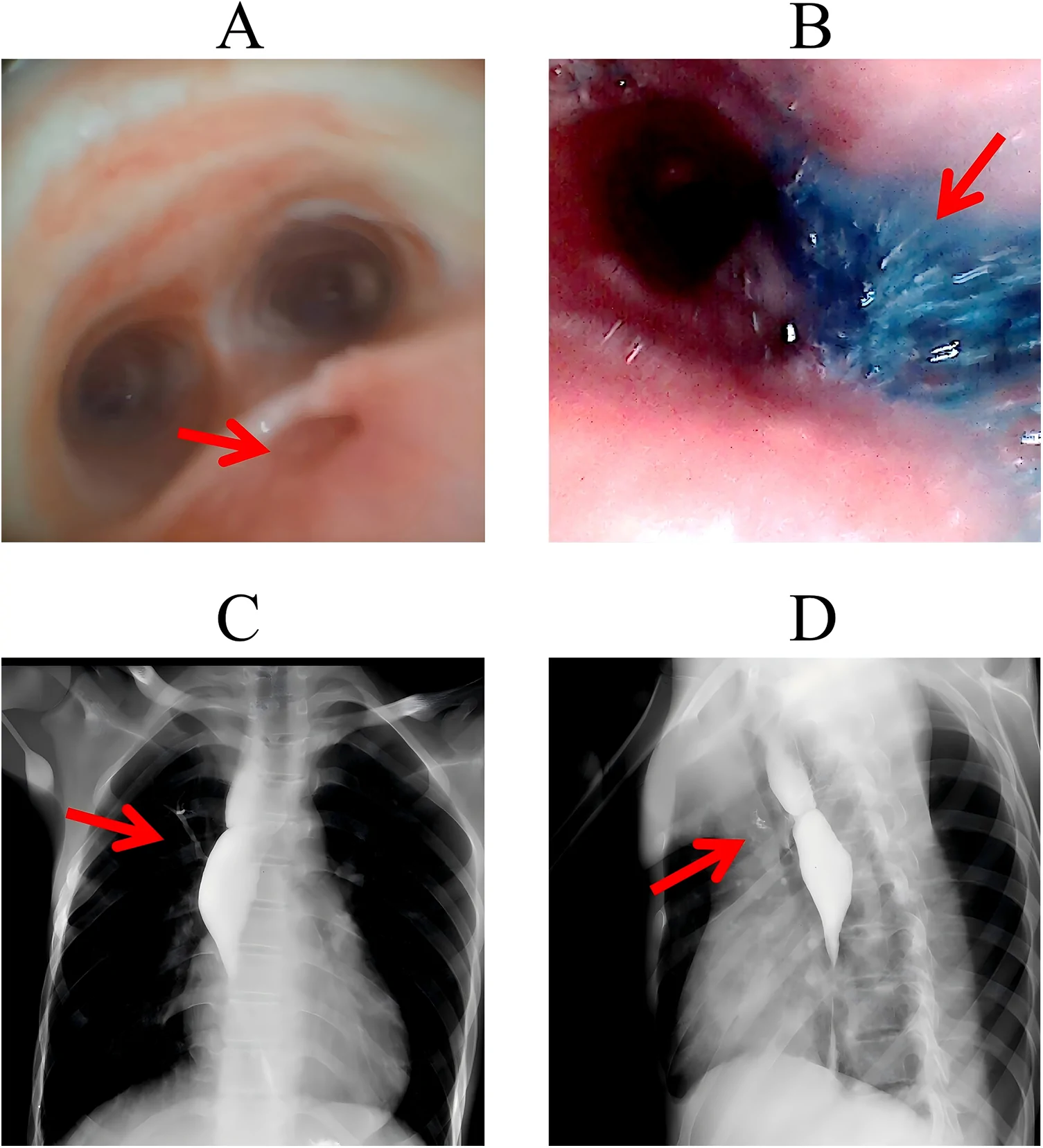
Fiberoptic bronchoscopy revealed the fistula located above the carina **(A)** after methylene blue was injected into the esophagus via a gastric tube, blue dye was observed leaking into the trachea from the fistula **(B)** esophagography demonstrated opacification of the right upper lobe bronchus following oral contrast administration **(C)**, further confirming the presence of tracheoesophageal fistula **(D)**.

**Figure 2:**
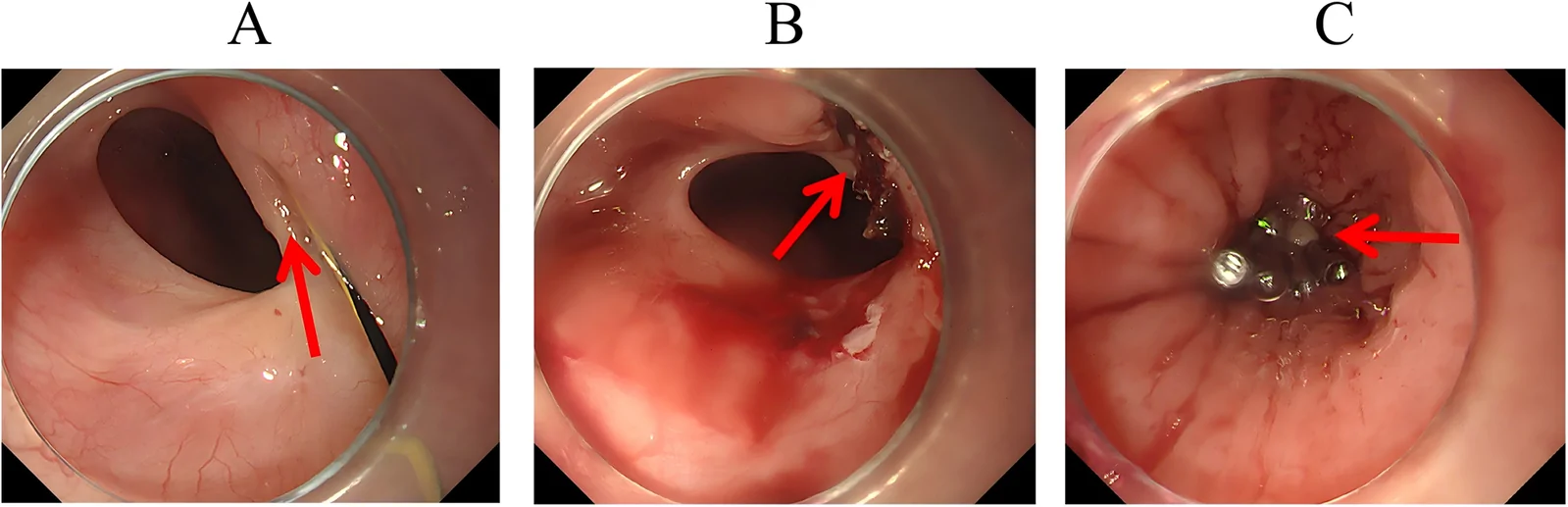
Under fiberoptic bronchoscopy, a zebra guidewire was inserted from the tracheal side through the fistula into the esophagus **(A)** under gastroscopy, the esophageal orifice of the fistula was cauterized using electrocoagulation to create a fresh wound surface **(B)**, followed by closure of the fistula with 4 titanium clips **(C).**

**Table 1:** Baseline clinical characteristics of the adolescent patient with rTEF.

Phase	Time Period	Duration	Core Clinical Events
Neonatal primary surgery stage	Postnatal Day 0—Postoperative Week 2	2 weeks	1. Diagnosed with Gross type III congenital esophageal atresia + tracheoesophageal fistula (TEF) 2. Open transthoracic esophageal anastomosis + TEF ligation repair on postnatal day 3(age 3 days) 3. Oral feeding started on POD10 4. Feeding choking occurred at postoperative week 2; recurrent TEF confirmed(age 2 weeks); converted to nasogastric tube feeding
Long-term follow-up blank period	Postoperative Week 2—January 2024	- 15 years 6 months	1. Stable growth after discharge, occasional mild choking 2. 3-month postoperative esophagography: false-negative fistula result (fistula shrinkage) 3. No subsequent regular hospital re-examination
First endoscopic intervention stage	January 2024(age 15 y 6 m)	1 month	1. Fever-induced severe pneumonia + feeding choking; readmitted 2. Imaging confirmed persistent recurrent TEF3. Antibiotic therapy; gastroscopy-guided TEF closure with 4 titanium clips 4. POD10 esophagography showed complete fistula healing; discharged
Second recurrent infection & reoperation stage	February 2025(age 16 y 7 m)	1 month	1. School cross-infection induced recurrent pneumonia, poor antibiotic response 2. Second open transthoracic TEF repair after family communication 3. Postoperative persistent cough, expectoration and choking; residual TEF confirmed by imaging 4. Improved with antibiotic therapy and nutritional support before discharge
Pre-admission observation period	March 2025—December 2025	9 months	Post-discharge recurrent respiratory infections, ineffective antibiotics and progressive malnutrition
Current admission stage	December 2025(age 17 y 6 m)	On admission	Readmitted after external antibiotic treatment for standardized MDT evaluation and definitive endoscopic repair in January 2026 (age 17 y 7 m)

Brief family-psychosocial history: Short paragraph noting unremarkable family medical history; long-term illness burden, school-absenteeism, and adolescent psychosocial stress related to chronic choking and recurrent pneumonia.

### Physical examination and diagnostic assessment

The patient was readmitted in December 2025 after clinical stabilization following antibiotic management at an external hospital. Physical examination findings were as follows: resting vital signs remained stable; peripheral oxygen saturation (SpO₂) under room air fluctuated between 93% and 95%. Mild tachypnea and audible stridor were noted during emotional agitation, while no obvious inspiratory retraction was observed at rest. Subcutaneous fat was markedly depleted secondary to long-standing malnutrition. A well-healed old surgical scar was visible on the right lateral chest wall.

Diagnostic reasoning was essential in this case, given that the patient's chronic choking and recurrent pneumonia could be attributed to tracheomalacia, gastro-esophageal reflux, or rTEF, thus requiring careful clinical evaluation and differential diagnosis. Isolated tracheomalacia may cause stridor and recurrent respiratory infections; however, flexible bronchoscopy identified a definite fistulous orifice, and extravasation of contrast medium on esophagography confirmed an abnormal communication between the esophagus and trachea, which cannot be explained by tracheomalacia alone. Aspiration secondary to gastro-esophageal reflux in the absence of a fistula was also considered. Nevertheless, the patient's choking episodes were closely correlated with oral intake, and direct endoscopic visualization of the fistula ruled out aspiration caused merely by reflux. Combined findings from chest CT, esophagography, and flexible bronchoscopy led to the final diagnosis of persistent rTEF complicated by diffuse tracheomalacia. Laboratory test results showed prealbumin 144.7 mg/L and C-reactive protein (CRP) 7.22 mg/L, with serum electrolytes within normal reference ranges. Nutritional risk screening was performed upon admission using the STRONGkids tool (score = 4) and NRS2002 scale (score = 3), consistent with a high nutritional risk classification.

## Interventions

### MDT and perioperative management

Given the patient's multiple preoperative problems including chronic respiratory inflammation, severe nutritional risk and adolescent psychological disturbances, an MDT group was established, consisting of thoracic surgeons, respiratory endoscopists, otorhinolaryngologists, anesthesiologists, gastroenterologists, clinical nutritionists, psychologists and rehabilitation therapists. According to the results of MDT consultation, the patient received preoperative treatments including piperacillin-tazobactam for anti-infection, omeprazole for acid suppression and anti-reflux, oxygen therapy, nebulization for sputum removal, combined parenteral and enteral nutrition, psychological counseling and pulmonary rehabilitation training. The nursing team assisted physicians in implementing and coordinating the treatment plan.

After exclusion of surgical contraindications, the patient underwent tracheoesophageal fistula repair via a transtracheal approach under general anesthesia with combined suspension laryngoscopy and flexible bronchoscopy on January 20, 2026. Three sutures were placed on the tracheal side of the fistula intraoperatively (Figure 3). The operation proceeded smoothly. Postoperative treatments included nil per os, gastrointestinal decompression, anti-infection therapy, acid suppression with omeprazole, oxygen inhalation, nebulization and combined parenteral and enteral nutrition. Esophagography and flexible bronchoscopy performed on postoperative day 10 confirmed complete fistula healing (Figure 4). The gastric tube was removed, and oral feeding was initiated gradually. The patient was discharged on postoperative day 14 with a body weight of 50.0 kg. One-month follow-up showed a body weight of 51.2 kg, normal oral feeding without choking, and no pulmonary infection or fistula recurrence.

**Figure 3:**
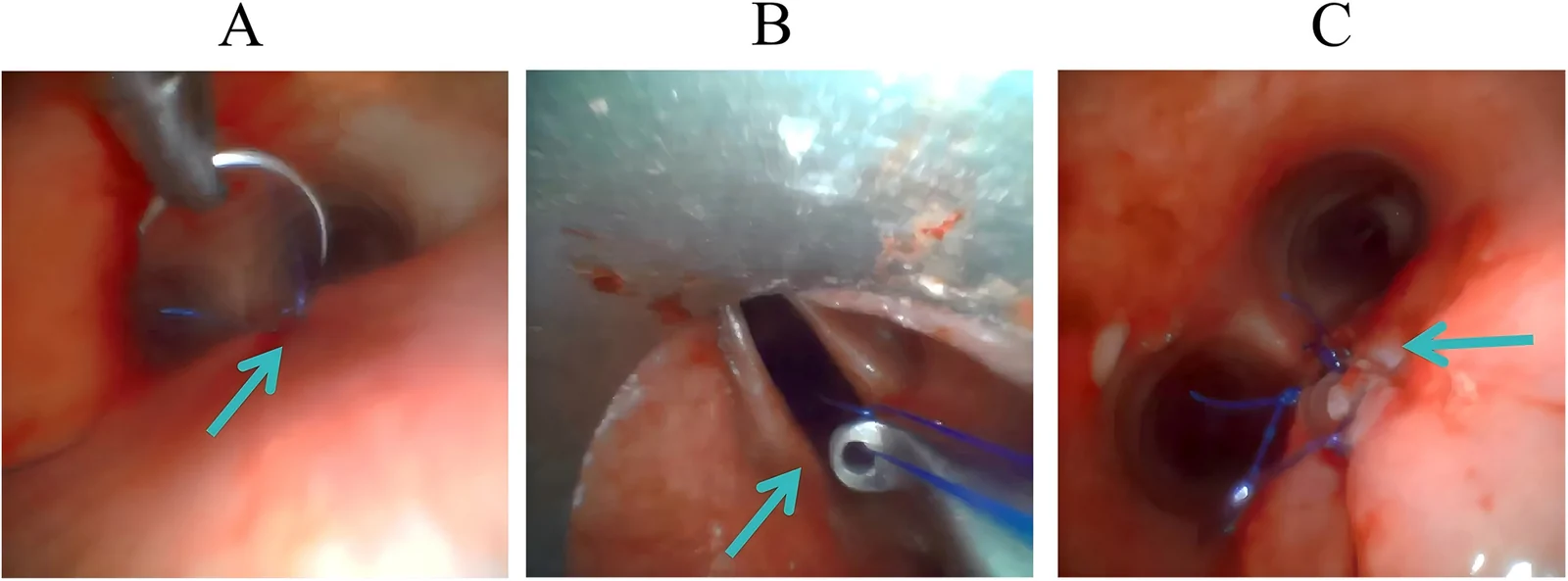
Endoscopic suturing of the fistula was performed using 4- 0 prolene suture under suspension laryngoscopy and fiberoptic bronchoscopy **(A)**, with extracorporeal knot tying **(B)** the fistula was completely closed with 3 sutures **(C).**

**Figure 4:**
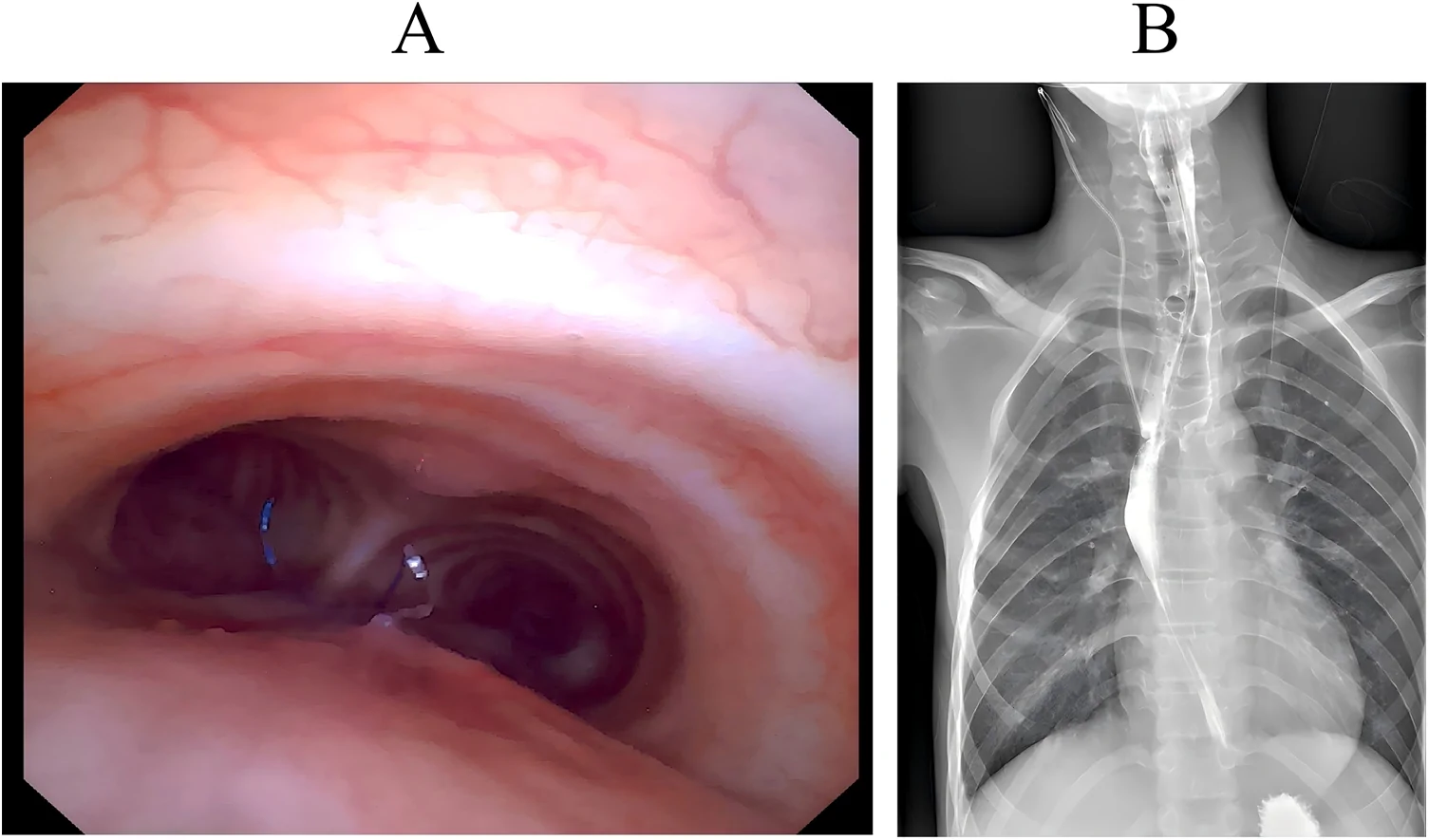
Fiberoptic bronchoscopy **(A)** and esophagography **(B)** confirmed satisfactory healing of the fistula.

## Postoperative nursing

### Psychological support nursing

Preoperatively, registered nurses collaborated with attending surgeons to illustrate fistula location, advantages of minimally invasive endoscopic surgery, postoperative discomfort and corresponding management strategies via anatomical diagrams. Psychologists guided the adolescent to actively express negative emotions to relieve perioperative anxiety and fear, facilitating smooth surgical performance and postoperative recovery (12, 13). Postoperatively, 15-minute bedside communication was implemented daily, and a family nursing log was established to train guardians to record fluid balance, sore throat, cough, reflux and emotional fluctuations, as well as participate in fundamental nursing care, improving family treatment adherence and self-care capacity (14).

### Refined perioperative respiratory nursing and management

The patient was maintained in a semi-recumbent position with head of bed elevated 35°–45° throughout the perioperative period, with moderate ambulation permitted to reduce gastroesophageal reflux and aspiration risk via gravitational effect. No airway catheter was placed intraoperatively or postoperatively; intraoperative ventilation was maintained via high-frequency jet ventilation under rigid laryngoscopy with lung-protective ventilation strategies. Nurses conducted continuous intraoperative rounds to monitor ventilation status, assisted anesthesiologists to guard against airway mucosal injury induced by mechanical stimulation from laryngoscopic instruments and high-frequency jet ventilation, and prevented complications such as atelectasis (15–17).

Continuous bedside SpO₂ monitoring was performed postoperatively. Persistent SpO₂ below 90% without obvious improvement after oxygen supplementation indicated severe airway obstruction requiring immediate physician notification. Nurses continuously monitored vocal changes, hoarseness and patient-reported throat pain or foreign body sensation. Postoperative low-flow oxygen inhalation (1–2 L/min) was administered via a thermostatic heated humidifier, with gas temperature maintained at 32–34 °C and relative humidity 32%–44% (18). Nebulization with budesonide combined with ipratropium bromide was delivered 2–3 times daily; post-nebulization chest percussion was performed to assist sputum expectoration, and effective coughing techniques were instructed (18).

Suction was performed on demand only based on clinical indicators including audible rales, SpO₂ < 92%, dyspnea and stridor, rather than fixed-interval blind suction. Pre-oxygenation for 1–2 min was delivered prior to suction, negative pressure was controlled at 0.02–0.04 MPa, single suction duration was limited to <15 s, and suction catheter diameter was less than half the airway inner diameter (19). During perioperative nil per os periods, chlorhexidine oral care was performed four times daily to reduce oropharyngeal pathogenic translocation and subsequent aspiration pneumonia.

### Staged evidence-based quantitative nutritional regimen

In accordance with the 2018 ESPEN Pediatric Nutrition Guidelines (20), the Schofield equation was applied to calculate resting energy expenditure (REE). Target nutritional goals were set as energy 35–40 kcal/(kg·d) and protein 1.8–2.0 g/(kg·d). Clinical nutrition specialists dynamically adjusted nutritional regimens perioperatively based on STRONGkids and NRS2002 screening results combined with serial prealbumin, CRP and serum electrolyte levels.

Postoperative days 1–3 (Total Parenteral Nutrition phase, 3 days): Given the patient's severe chronic malnutrition and high refeeding-syndrome risk, amino acids were initiated at 1.3 g/kg/day rather than immediately reaching the full ESPEN-recommended target of 1.8–2.0 g/kg/day, to prevent life-threatening electrolyte disturbance and hepatic metabolic overload. Protein provision was planned to be progressively up-titrated during the enteral-parenteral transition phase after stable biochemical monitoring results were obtained. Total parenteral nutrition was infused uniformly via central venous access. Daily monitoring of blood glucose, electrolytes and fluid balance revealed no significant abnormalities. Serum CRP decreased from admission 7.22 mg/L to 0.35 mg/L on postoperative day 3.

Postoperative days 4–9 (Combined enteral and parenteral nutrition transition phase, 6 days): Enteral nutrition was initiated after recovery of gastrointestinal function with an initial pump rate of 25 mL/h. The infusion rate was titrated upward by 10 mL/h every 2 days in the absence of abdominal distension, vomiting, gastric residual or gastroesophageal reflux. Nurses coordinated with nutrition specialists to reduce infusion speed upon feeding intolerance, while parenteral nutrition volume was gradually tapered. Full enteral nutrition was achieved on postoperative day 9, with serum prealbumin elevated to 271 mg/L.

Postoperative day 10 (Oral feeding transition phase): Gastric tube removal was performed after imaging confirmed complete fistula healing. Oral intake was advanced gradually from liquid to semi-liquid, soft food and regular diet with small, frequent meals. The patient was discharged on postoperative day 14 with body weight 50.0 kg, representing a 0.8 kg weight gain compared with admission, realizing quantitative correlation between nursing interventions, body weight and feeding progression.

### Discharge education and follow-up

After discharge, the patient was instructed to maintain small, frequent meals, avoid hard, cold and irritant foods, remain seated during feeding and maintain upright posture for over 30 min postprandially to prevent reflux. Home-based respiratory training including pursed-lip breathing and abdominal breathing was recommended; crowded public spaces, dust and irritant gas exposure were restricted to reduce respiratory infection risk. Mild physical activity such as walking and jogging was permitted, while high-intensity contact sports and chest trauma were strictly prohibited (21, 22). Immediate hospital consultation was mandated upon occurrence of persistent choking with liquid intake, unexplained fever complicated by cough and expectoration, progressive dysphagia or recurrent pneumonia within a short period.

Structured follow-up schedule was established:

1 month postoperatively: Assessment of height, weight, blood routine, prealbumin and chest CT;

3 months postoperatively: esophagography (upper gastrointestinal contrast study), fiberoptic bronchoscopy, pulmonary function testing and nutritional evaluation;

6 and 12 months postoperatively: Selective esophagography or fiberoptic bronchoscopy plus nutritional risk screening according to clinical status;

Annual follow-up beyond 1 year postoperatively, with a lifelong follow-up file created for the patient.

## Discussion

Recurrent tracheoesophageal fistula represents a severe long-term complication after primary CEA repair, with higher clinical complexity in adolescent patients with multiple prior failed surgical interventions. Current therapeutic options for rTEF can be divided into surgical and endoscopic approaches. Open transthoracic repair has long been the historical standard for rTEF (23, 24), offering direct visualization of the fistula and mediastinal tissues to enable thorough dissection and multilayer closure, yet it is associated with substantial morbidity including recurrent laryngeal nerve injury, chylothorax, esophageal stricture, and prolonged hospital stay (25). Pre-existing dense intrathoracic adhesions in patients with previous thoracic surgeries raise operative complexity and iatrogenic injury risk. This patient experienced two failed open transthoracic repairs, demonstrating the limitations of repeated open surgery for complex adolescent rTEF. Thoracoscopic transthoracic repair represents its minimally-invasive modification, lowering chest-wall trauma and postoperative pain while preserving fistula dissection and closure capacity from the esophageal side (26).Even so, this approach still requires thoracic cavity access, with risks of pneumothorax, pleural adhesions, and conversion to open surgery; pleural symphysis after multiple prior thoracic procedures may render thoracoscopic access unsafe.

Endoscopic approaches have emerged as alternative strategies for rTEF, particularly in patients who are poor candidates for repeat thoracic surgery. These include:(i) Endoscopic titanium clip closure (27, 28). As performed in this patient's first endoscopic intervention, titanium clips are deployed to seal the esophageal orifice of the fistula. This technique is technically straightforward and can be performed in a single session. However, as demonstrated in this case, clip closure of only the esophageal orifice carries a high recurrence risk because tracheal secretions continue to perfuse the fistula tract from the unclosed tracheal side. Clip migration, inadequate tissue apposition, and inability to close wide fistula defects are additional limitations.(ii) Laser ablation. Endoscopic laser ablation (29, 30) has been reported for small rTEF. The laser is applied to the epithelial lining of the fistula to induce thermal injury and subsequent closure via granulation-tissue formation. Its reported advantages include minimal trauma and the ability to manage fistulas that are mechanically inaccessible. Nevertheless, laser ablation carries relevant risks: thermal injury to adjacent tracheal and esophageal mucosa may result in tracheal stenosis, esophageal stricture, or even fistula enlargement. Success rates vary widely, multiple treatment sessions are often required, and adjunctive therapies such as biological glue and clips are frequently needed (31). (iii) Endoscopic suturing under combined suspension laryngoscopy and flexible bronchoscopy. This approach enables direct suture closure of the fistula from the tracheal side, which is fundamentally different from clip closure or laser ablation. By closing the tracheal orifice, the technique blocks the continuous perfusion of tracheal secretions into the fistula tract — a key mechanism underlying recurrence after esophageal-side-only closure. The technique avoids thoracic entry entirely, making it particularly valuable for patients with multiple prior thoracic surgeries and dense pleural adhesions (7, 8).

Three core strengths characterize our nursing management strategy: (1) individualized nursing planning guided by multidisciplinary team collaboration; (2) standardized sputum suction and humidification protocols enabling refined airway management; (3) staged quantitative nutritional support formulated in compliance with ESPEN guidelines (20). Favorable clinical outcomes including absence of postoperative complications, complete fistula closure and significant weight gain validate the efficacy of this nursing framework. Several limitations should be acknowledged: this single-case report restricts generalizability to all rTEF patients, and long-term durability of endoscopic fistula repair requires extended follow-up. Further large-cohort long-term observational studies are warranted to validate the proposed nursing strategies.

## Patient perspective

One month after discharge, the adolescent shared his perioperative experience: ‘I have suffered from choking with liquid intake for 17 years, and two prior surgeries failed. I was deeply anxious that this third intervention might also not work. Pre-operatively, nurses used anatomical diagrams to explain endoscopic minimally-invasive surgery, and psychologists guided breathing relaxation exercises to reduce my anxiety. Post-operatively, staged enteral feeding relieved hunger discomfort, and daily bedside communication helped me endure throat pain. Now I can safely take soft food without choking, my weight has increased, and I can socialize normally with classmates. This whole-course care eased my physical wound-healing burden as well as long-term psychological distress from chronic disease. I will comply with scheduled long-term follow-up.’

## Conclusion

For multiply-operated adolescent rTEF patients, multidisciplinary-team full-cycle perioperative care integrating psychological intervention, refined airway management and staged quantitative nutrition support is recommended when dual-endoscopic fistula repair is planned. Clinicians should be alerted that incompletely healed rTEF may remain clinically silent with only mild intermittent symptoms for many years; structured long-term follow-up is therefore mandatory for patients after primary esophageal atresia repair, even when initial postoperative imaging appears normal.

## Data Availability

All de-identified clinical raw data (laboratory results, endoscopic images, esophagography films, nursing records and follow-up data) of this adolescent rTEF case are securely preserved in the offline Electronic Medical Record Databaseof Children’s Hospital, Zhejiang University School of Medicine. The full anonymized dataset can be reasonably obtained upon valid academic application to the corresponding author (laxiajie@zju.edu.cn).
